# A New Strategy Toward B Cell-Based Cancer Vaccines by Active Immunization With Mimotopes of Immune Checkpoint Inhibitors

**DOI:** 10.3389/fimmu.2020.00895

**Published:** 2020-05-27

**Authors:** Joshua Tobias, Claire Battin, Annika De Sousa Linhares, Michael Lebens, Karin Baier, Katharina Ambroz, Mirjana Drinić, Sandra Högler, Aleksandra Inic-Kanada, Erika Garner-Spitzer, Matthias Preusser, Lukas Kenner, Michael Kundi, Christoph C. Zielinski, Peter Steinberger, Ursula Wiedermann

**Affiliations:** ^1^Center for Pathophysiology, Infectiology and Immunology, Institute of Specific Prophylaxis and Tropical Medicine, Medical University of Vienna, Vienna, Austria; ^2^Division of Immune Receptors and T Cell Activation, Center for Pathophysiology, Infectiology and Immunology, Institute of Immunology, Medical University of Vienna, Vienna, Austria; ^3^Department of Microbiology and Immunology, Institute of Biomedicine, University of Gothenburg Vaccine Research Institute (GUVAX), University of Gothenburg, Göteborg, Sweden; ^4^Unit of Laboratory Animal Pathology, Institute of Pathology, University of Veterinary Medicine Vienna, Vienna, Austria; ^5^Division of Oncology, Department of Medicine I, Medical University of Vienna, Vienna, Austria; ^6^Department of Experimental Pathology, Medical University of Vienna, Vienna, Austria; ^7^Department of Environmental Health, Center for Public Health, Medical University of Vienna, Vienna, Austria; ^8^Vienna Cancer Center (VCC), Medical University Vienna, and Vienna Hospital Association, Vienna, Austria

**Keywords:** mimotopes, immune checkpoint inhibitors, active immunization, cancer therapy, Her-2/neu, combination therapy

## Abstract

Therapeutic monoclonal antibodies (mAbs), targeting tumor antigens, or immune checkpoints, have demonstrated a remarkable anti-tumor effect against various malignancies. However, high costs for mono- or combination therapies, associated with adverse effects or possible development of resistance in some patients, warrant further development and modification to gain more flexibility for this immunotherapy approach. An attractive alternative to passive immunization with therapeutic antibodies might be active immunization with mimotopes (B-cell peptides) representing the mAbs' binding epitopes, to activate the patient's own anti-tumor immune response following immunization. Here, we identified and examined the feasibility of inducing anti-tumor effects *in vivo* following active immunization with a mimotope of the immune checkpoint programmed cell death 1 (PD1), alone or in combination with a Her-2/neu B-cell peptide vaccine. Overlapping peptides spanning the extracellular domains of human PD1 (hPD1) were used to identify hPD1-derived mimotopes, using the therapeutic mAb Nivolumab as a proof of concept. Additionally, for *in vivo* evaluation in a tumor mouse model, a mouse PD1 (mPD1)-derived mimotope was identified using an anti-mPD1 mAb with mPD1/mPDL-1 blocking capacity. The identified mimotopes were characterized by *in vitro* assays, including a reporter cell-based assay, and their anti-tumor effects were evaluated in a syngeneic tumor mouse model stably expressing human Her-2/neu. The identified PD1-derived mimotopes were shown to significantly block the mAbs' capacity in inhibiting the respective PD1/PD-L1 interactions. A significant reduction in tumor growth *in vivo* was observed following active immunization with the mPD1-derived mimotope, associated with a significant reduction in proliferation and increased apoptotic rates in the tumors. Particularly, combined vaccination with the mPD1-derived mimotope and a multiple B-cell epitope Her-2/neu vaccine potentiated the vaccine's anti-tumor effect. Our results suggest active immunization with mimotopes of immune checkpoint inhibitors either as monotherapy or as combination therapy with tumor-specific vaccines, as a new strategy for cancer treatment.

## Introduction

Multiple lines of preclinical and clinical evidence have shown that tumors can evade the immune system by expressing surface ligands, which engage co-inhibitory receptors on tumor-specific T cells resulting in immune tolerance (1, 2). The interaction between the immune checkpoint programmed cell death 1 (PD1), a co-inhibitory receptor on T cells, with its ligand PD-L1, plays a central role in this approach. PD-L1 is expressed by a multitude of immune cells and also on some cancer cells, and the interaction between PD1 and PD-L1 results in activation of PD1 and, in turn, attenuation of T-cell activation (3–5).

Antibody engineering has played a major role in the development of therapeutic monoclonal antibodies (mAbs) against cancer or immune structures (6, 7). Among such mAbs, those targeting PD1 (e.g., Nivolumab, Pembrolizumab) or PD-L1 (e.g., Atezolizumab, Durvalumab), i.e., immune checkpoint inhibitors (ICIs), are considered a significant milestone and hold a tremendous promise for the treatment of diverse solid tumor types (8, 9). Although with impressive therapeutic efficacy, potential adverse effects, frequent applications in relatively short time intervals, and the cost intensiveness as a result of the long duration of treatments with mAbs may pose significant disadvantages (10–15). Such drawbacks may be circumvented using respective B-cell peptides, which represent the mAbs' binding epitope, i.e., mimotope, to induce the patient's own anti-tumor immune responses following active immunization. The use of mimotopes for vaccines has become a promising strategy both for infectious diseases and diagnostics (16–18) as well as for cancer therapy (19), and the functionality of mimotope-based cancer vaccines has also previously been shown in an experimental cancer model (20). In this respect, we earlier described a B-cell multi-peptide vaccine against the antigen Her-2/neu, covering the binding epitope of Trastuzumab. This vaccine showed an excellent safety profile and strong immunogenicity in patients with Her-2-positive metastatic breast cancer (21, 22), associated with clinical responses in gastric cancer patients in a phase I/b clinical studies (23, 24).

In this study, we aimed to identify mimotopes derived from PD1 (human “hPD1” and mouse PD1 “mPD1”) and characterize their PD1/PD-L1 blockade capacity using different *in vitro* assays, including reporter T cells expressing PD1 for functionality testing. Importantly, *in vivo* evaluation of the mPD1-derived mimotope's anti-tumor effect as a monovalent vaccine and in combination with a Her-2/neu vaccine following active immunization was shown in a syngeneic tumor mouse model with tumors expressing human Her-2/neu.

## Materials and Methods

The generation and expression of overlapping peptides, detection, and characterization (by solid phase-based assays) of the identified mimotopes, sequence analysis, peptide synthesis, ELISA, and inhibition ELISA are detailed in the Supplementary Materials and Methods.

### Bacteria, Cell Lines, and Growth Conditions

The *Escherichia coli* strain BL21 (New England Biolabs) was used in this study for expression of overlapping peptides and grown in LB medium supplemented with Kanamycin (50 μg/ml).

The Jurkat E6.1 NF-κB::eGFP reporter T cell line and the K562 stimulator cell line were cultured as described previously (25). JE6.1 NF-κB::eGFP reporter cells expressing human PD1 (hPD1) or mouse PD1 (mPD1) have been previously described (26). T-cell stimulator cells, based on the K562 cell line (short designation in this work: K562S), were generated by retrovirally transducing a CD5L–OKT3scFv–CD14 construct encoding an anti-human CD3 single-chain fragment fused to human CD14 (27). K562S stimulate primary human T cells and T cell lines by ligating their TCR–CD3 complex. In order to separate stimulator cells from reporter cells, K562S were engineered to constitutively express a red fluorescent protein (RFP). K562S–RFP cells expressing high levels of human PD-L1 (hPD-L1) were generated via retroviral transduction. Single-cell clones were established to assure homogenous and comparable expression of the respective molecules. To confirm cell surface expression of respective molecules, the following PE-conjugated antibodies from Biolegend (San Diego, CA, USA) were used: hPD1 (EH12.2H7), mPD1 (29F.1A12), and hPD-L1 (29E.2A3). Membrane-bound anti-CD3 fragment on K562S cells was detected with a PE-conjugated goat-anti-mouse IgG (H + L) antibody (Jackson ImmunoResearch, West Grove, PA, USA). Acquisition of flow cytometry data was performed using FACS Calibur with CellQuest software (both from BD Biosciences, San Jose, CA, USA). Data were analyzed using FlowJo software (version 10.0.8.; Tree Star, Ashland, OR, USA) and Graphpad Prism (version 5; GraphPad Software, Inc., La Jolla, CA, USA).

D2F2/E2 cells, a BALB/c mouse cell line derived from a spontaneous mammary tumor also stably expressing human breast-associated tumor antigen Her-2/neu, were kindly provided by Prof. Wei-Zen Wei (Karmanos Cancer Institute, Wayne State University School of Medicine, Detroit, Michigan, USA). The cells were maintained in high-glucose DMEM, supplemented with 100 U/ml of penicillin, 100 μg/ml of streptomycin, 10% FBS, 10% NCTC 109, 1% non-essential amino acids, and 5% sodium bicarbonate.

### Inhibition ELISA

Inhibition ELISA systems were established and employed to evaluate the (1) capacity of the identified mimotopes in inhibiting the binding of the anti-hPD1 or the rat anti-mPD1 mAbs to recombinant hPD1 or mPD1 HIS-tagged proteins (in PBS; R&D Systems, Minneapolis, MN, USA) in a solid-phase ELISA, respectively, and (2) capacity of JT–mPD1 rabbit IgG in inhibiting the interaction between recombinant mPD1–HIS/mPD–L1–Fc chimera.

Evaluation of the examined mimotopes' capacity in inhibiting the binding of the anti-hPD1 mAb Nivolumab (2 ng/ml) or rat anti-mPD1 (200 ng/ml) mAb to recombinant HIS-tagged mPD1 or hPD1 proteins (R&D Systems, Minneapolis, MN, USA), respectively, was carried out as follows. The recombinant proteins were used for coating MAXISORP (NUNC) plates (0.1 μg/well), and the coated wells were blocked with PBS–skim milk 2%. The mAbs preincubated with different concentrations of the examined hPD1 or mPD1-derived mimotopes were added into the coated wells. As negative control a 15-mer peptide (PHQGQHIGEMSFLQH) was also included in the assays. Bound mAbs to the coated recombinant proteins were detected using mouse anti-rat IgG (against anti-mPD1 mAb; HRP-conjugated; Jackson ImmunoResearch, Cambridgeshire, UK) or mouse anti-human IgG (Fc; against the anti-hPD1 mAb; HRP-conjugated; SouthernBiotech, Birmingham, AL, USA) as secondary antibodies. Bound secondary antibodies were subsequently detected by staining with TMB substrate solution (Invitrogen, Vienna, Austria), and the ELISA OD values were measured at 450 vs. 630 nm (Tecan Spark™ 10 M Multimode Plate Reader).

For the inhibition ELISA to evaluate the capacity of rabbit IgG against JT–mPD1 in inhibiting the interaction between recombinant mPD1–HIS and mPD–L1–Fc, different examined rabbit IgG concentrations were added to the wells coated with recombinant mPD1–HIS (1 μg/ml), followed by addition of recombinant mPD–L1–Fc (5 μg/ml). Bound mPD-L1-Fc to the coated mPD1-HIS was detected using mouse anti-human IgG (Fc; HRP-conjugated) as a secondary antibody. The bound HRP-conjugated secondary antibody was subsequently detected as above.

### Binding and Functional Assays Using Jurkat Reporter Cells Expressing Human or Mouse PD1

The mimotopes of the anti-hPD1 mAb Nivolumab (JT–N1, JT–N2, alone or in combination) and of anti-mPD1 mAb (JT–mPD1), as well as the negative control mimotope (PHQGQHIGEMSFLQH), in 1 × PBS, were incubated at the indicated concentrations with the corresponding mAbs (in 1 × PBS, 0.5% BSA, 0.005% sodium azide) for 60 min at room temperature. Per condition, 1 × 10^5^ Jurkat reporter cells expressing high levels of human PD1 (hPD1) or mPD1 were added and incubated for 30 min at 4°C. Binding of the anti-hPD1 (50 ng/ml) and anti-mPD1 mAb (10 ng/ml) was detected with APC-conjugated goat-anti-human (Fc) antibodies and APC-conjugated goat anti-rat (Fc) antibodies (both from Jackson ImmunoResearch), respectively. Samples were analyzed via flow cytometry, and mean and standard deviation of the geometric mean of fluorescence intensity (gMFI) of the viable population of reporter cells were determined (25).

For functional assays, different examined concentrations of the anti-hPD1 mAb mimotope JT–N1 and the mAb (150 ng/ml) were preincubated for 60 min at room temperature. Then, the previously described reporter cells expressing human PD1 (5 × 10^4^ cells/well) were added and co-cultured with stimulator cells co-expressing a membrane-bound CD3–antibody fragment and human PD–L1 (1 × 10^4^ cells/well) for 24 h at 37°C in 5% CO_2_ atmosphere (26). Cells were then harvested, and eGFP expression was analyzed via flow cytometry. Mean and standard deviation of the gMFI of the viable population of reporter cells (RFP+ stimulator cells were excluded) were determined. Each experiment was performed in duplicates, unless stated otherwise.

### Syngeneic Tumor Mouse Model for *in vivo* Anti-tumor Evaluation

#### Passive Immunization

For evaluation of anti-tumor effect of rabbit IgG generated against JT–mPD1, female BALB/c mice (6–8 weeks of age at the time of delivery; Charles River, Germany) were used. The experiment consisted of four groups of mice (*n* = 8): naïve (injected with PBS), sham treated (injected with IgG from control rabbits), injected with IgG from JT–mPD1-immunized rabbits, or with anti-mouse PD1 mAb (clone 29F.1A12). Mice were bled and injected i.p. with the above mentioned antibodies on day 0, and 1 day later 2 × 10^6^ BALB/c mice-derived mammary carcinoma cells stably expressing human Her-2/neu cells (D2F2/E2) were injected s.c. into the mice left flank. Thereafter, the grafted mice received two more injections (i.p.) with the antibodies on days 6 and 11. Two weeks after the grafting, the mice were sacrificed, the tumors were explanted, and their weight was measured (**Figure 3B**).

#### Active Immunization

In a second setting involving active immunization of the mice with JT-mPD1 (50 μg/dose), or Her-Vaxx (22, 23) (Her-2/neu multiple B-cell peptide vaccine; 25 μg/dose) or in combination, mice were bled before the first immunization, and immunized s.c. five times with 2-week intervals. One week after the fifth immunization, 2 × 10^6^ D2F2/E2 cells were grafted as above, followed by an additional (sixth) immunization 1 week after the cells injection. Two weeks after the grafting, the mice were sacrificed, the tumors were explanted, and their weight was measured (**Figure 4A**).

Pilot studies, involving testing different numbers of D2F2/E2 cells and measurements of the developed tumors (based on volume and weight) at different post-grafting time points, were carried out and provided the basis for the above schedules of the immunization experiments in this study.

The experiments were approved by the Animal Experimentation Committee of the Medical University of Vienna and the University of Veterinary Medicine as well as by the Austrian Federal Ministry of Science and Research (BMWF-66.009/0136-WF/V/3b/2017).

### Immunohistochemistry and Histological Analysis

Tumor-derived tissues were formalin fixed, paraffin-embedded (FFPE), and 3 μm sections were stained for Ki67 and cleaved caspase-3. Briefly, the de-waxed sections were heated in citrate buffer (pH 6.0) for antigen retrieval. The endogenous peroxidase was blocked in 3% hydrogen peroxide in PBS. Additional blocking steps were performed using the Avidin/Biotin blocking kit (Vector laboratories), Super block (Empire Genomics), and mouse block (Empire Genomics). The primary antibodies Ki67 (No. 12202; Cell Signaling) and cleaved caspase-3 (No. 9661; Cell Signaling) were applied in PBS + 1% BSA at 4°C overnight. The IDetect Super Stain System HRP (Empire Genomics) was used for further steps, and the signal was visualized with 3-Amino-9-Ethylcarbazole (BD Pharmingen) followed by a counterstaining with hematoxylin. An IgG control (No. 3900; Cell Signaling) was used for both antibodies as negative control.

Sections were evaluated with an Olympus BX-53 microscope (Olympus, Tokyo, Japan), and one to three images per tumor, depending on tumor size, were taken with an Olympus DP-26 camera (Olympus, Tokyo, Japan). The open source software Fiji by ImageJ was used to analyze the images and quantify the stained areas in the images (28).

### Statistical Analysis

Binding levels (gMFI) and OD values from ELISA were log-transformed to account for the skewed distribution, and to stabilize variances, binding capacity data, and tumor volumes were square-root transformed. Comparison against a control treatment was performed by analysis of variance (ANOVA) with subsequent Bonferroni–Holm-corrected contrasts for conditions with more than two groups. Investigation of combined inhibitory activity of JT–N1-3 was done using Loewe additivity as a reference. For this purpose, for each result of the combined mimotopes, the sum C_A_/EC_A_ + C_B_/EC_B_ was computed, where C_A_ and C_B_ denote the concentration of mimotopes A and B in the combination, respectively, and EC_A_ and EC_B_ are the respective concentrations of these mimotopes expected to result in the same inhibition as found in the combined experiment. These expected concentrations were derived from the concentration-inhibition curves for each mimotope alone. This was done for all concentrations that exhibit inhibition and tested for each mimotope combination separately against the reference value of 1 by Student's *t*-tests. For the IHC data on CC3 and Ki67, log-transformed values were subjected to a mixed model ANOVA with animals as random factor nested within the group factor, because a variable number of slides was available for each animal. All analyses were done by Stata 13.1 (StataCorp, TX, USA), and graphs were prepared by GraphPad Prism 7 (GraphPad Software, CA, USA). For all statistical tests, a value of *p* < 0.05 was considered significant.

## Results

### Identification of hPD1- and mPD1-Derived Mimotopes

**Figure 1:**
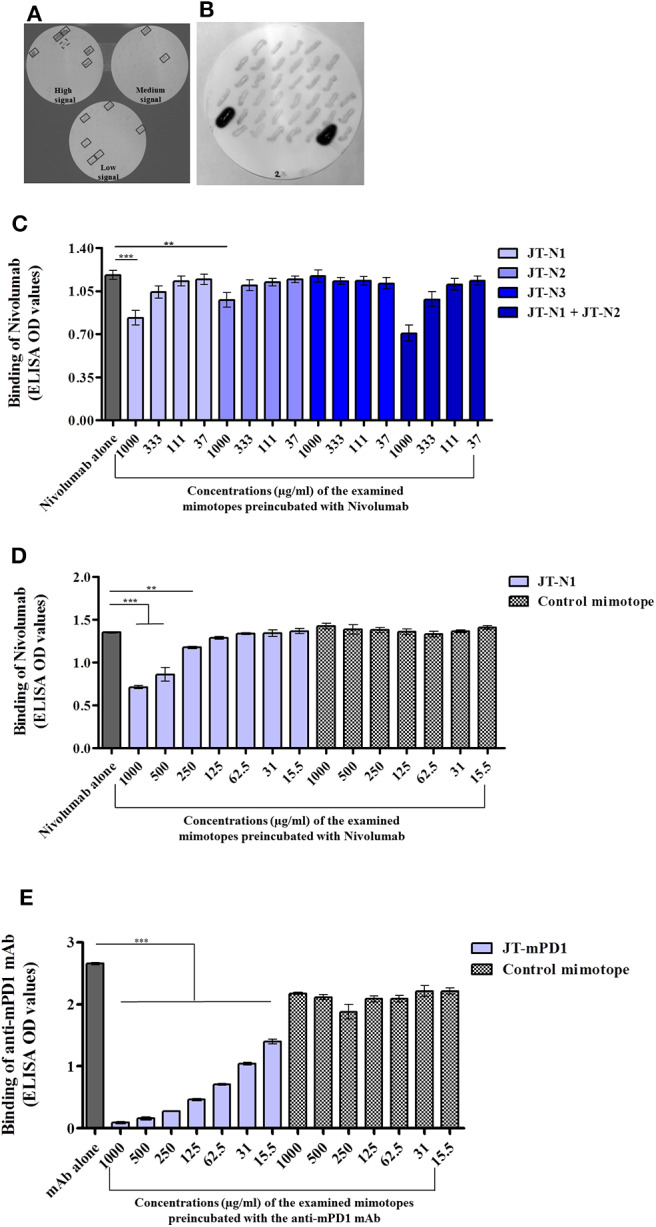
Identification of the mimotopes of the anti-human programmed cell death 1 (hPD1) mAb Nivolumab and anti-mouse PD1 (mPD1) monoclonal antibody (mAb), and examination of their specificity. Colony blot assay was applied on clones of *Escherichia coli* individually expressing overlapping peptides spanning the entire extracellular domain of hPD1 **(A)** or mPD1 **(B)** with anti-hPD1 and anti-mPD1 mAbs used for detection, respectively, as described in the Materials and Methods section. In the colony blot assay with anti-hPD1 mAb, the detected clones are boxed with solid line. One positive clone with failed sequencing is boxed with a broken line. Capacity of the identified mimotopes JT–N1, JT–N2, and JT–N3 **(C,D)** and JT–mPD1 **(E)** with comparison to a control mimotope, in inhibiting the binding of anti-hPD1 and anti-mPD1 mAbs to recombinant hPD1 or mPD1 proteins, respectively, is shown. Recombinant hPD1 or mPD1 proteins were used for coating in a solid phase-based assay (ELISA), and binding of the respective mAbs to the coated proteins was evaluated alone or after preincubation with different examined concentrations of the respective mimotopes. The results are representative of at least two repeated experiments. Significant differences are indicated by asterisks (**P < 0.01, ***P < 0.001).

Libraries of expression vectors individually encoding overlapping peptides (15-mers) spanning the entire extracellular domain of hPD1 and mPD1 were used, as described in the Materials and Methods section. Screening clones of *E. coli* expressing the individual overlapping peptides, with anti-hPD1 mAb Nivolumab, used as a model in this study, revealed several clones with signals at three different intensity levels (boxed; Figure 1A). The detected clones were shown to express the overlapping peptides PGWFLDSPDRPWNPP, FLDSPDRPWNPPTFS, and SPDRPWNPPTFSPA, corresponding to the positions 21–35, 24–38, and 27–41 on hPD1, designated as JT–N1, JT–N2, and JT–N3, respectively.

As a proof for *in vivo* anti-tumor evaluation, in mice, a similar strategy of mimotope identification was also employed on mPD1 using an anti-mPD1 mAb with blocking capacity (clone 29F.1A12). As shown in Figure 1B, the anti-mPD1 mAb strongly reacted with two clones, and sequence analysis of the overlapping peptides in the two detected positive clones indicated that both clones expressed the same peptide with the sequence ISLHPKAKIEESPGA (JT–mPD1) corresponding to amino acid residues 126–140 of mPD1.

The capacity of the identified mimotopes to inhibit the binding of the anti-hPD1 mAb Nivolumab to recombinant hPD1 protein was examined in inhibition ELISA, by preincubating the mAb with the mimotope at different concentrations. As shown in Figure 1C, the mimotope JT–N1 dose-dependently exhibited the strongest inhibition, followed by the mimotope JT–N2 but not by JT–N3. Testing the combination of JT–N1 and JT–N2 in the assay only marginally increased the binding inhibition compared to the inhibition observed by JT–N1 alone (Figure 1C), suggesting that among the identified mimotopes, JT–N1 is the most specific mimotope of Nivolumab.

The observed specific inhibitory capacity of the selected mimotope JT–N1 was further examined in inhibition ELISA, also including a 15-mer control mimotope (PHQGQHIGEMSFLQH). As shown in Figure 1D, the control mimotope did not inhibit the binding of Nivolumab, verifying the specificity of the mimotope JT–N1 in inhibiting the binding of the mAb in a dose-dependent manner.

The capacity of the mimotope JT–mPD1 in inhibiting the binding of the corresponding mAb to recombinant mPD1 was also examined in inhibition ELISA. As shown in Figure 1E, preincubation of the anti-mPD1 mAb with the mimotope dose-dependently, and potently inhibited the binding of the mAb to recombinant mPD1 used in the assay. The specificity of the mimotope was further verified by showing no binding inhibition of the mAb after its preincubation with the control mimotope in the same assay (Figure 1E).

### The Identified Mimotopes From hPD1 and mPD1 Inhibit the Interaction Between the Corresponding PD1 and PD–L1 *in vitro*

**Figure 2:**
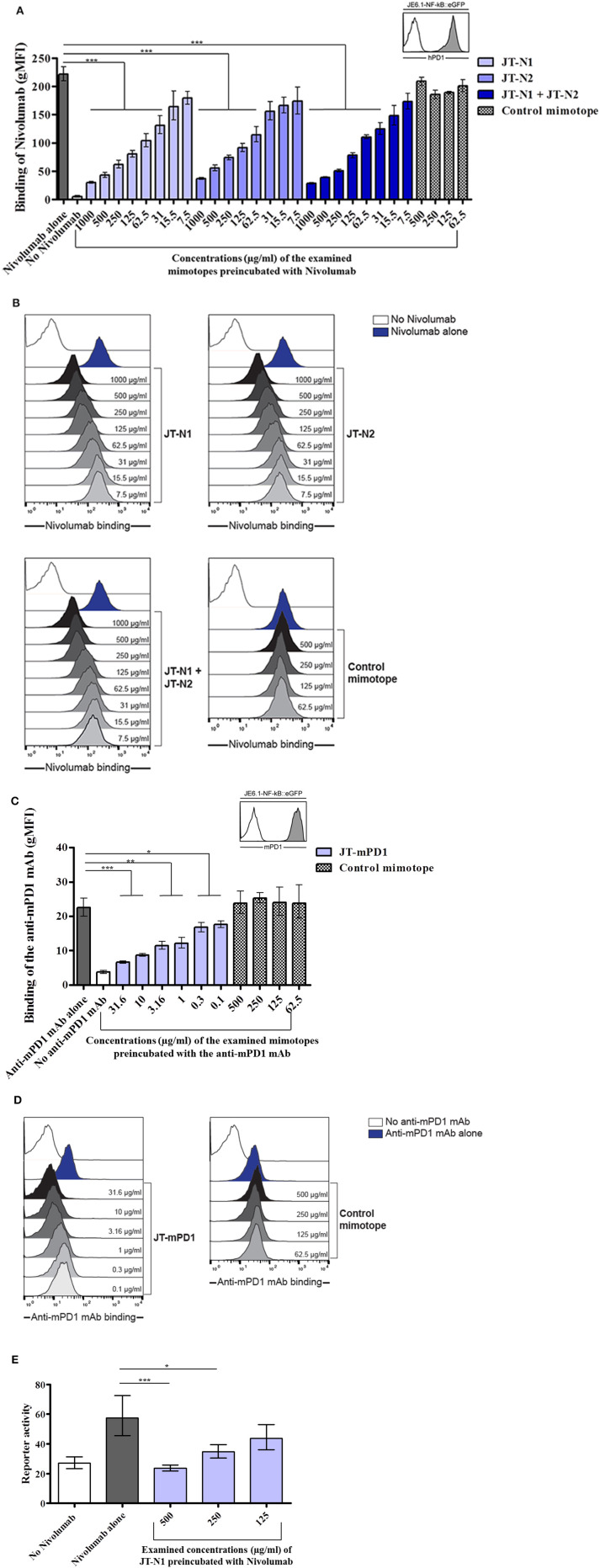
Examination of the specificity of the mimotopes JT–N1, JT–N2, and JT–mPD1 in cellular assays. **(A)** Jurkat T cells expressing high levels of hPD1 were used in a binding assay to examine the binding of anti-hPD1 mAb Nivolumab (50 ng/ml) alone or after preincubation with different concentrations of JT–N1, JT–N2, combination of both mimotopes, and a control mimotope. The inset shows reactivity of an antibody to hPD1 on Jurkat–hPD1 (gray histogram) and control Jurkat cells (open histogram) *n* = 4 for each data point. **(B)** Flow cytometry histograms of a representative experiment are shown. Open histogram, no Nivolumab; blue histogram, binding of Nivolumab alone; gray histograms, titration of JT–N1, JT–N2, combination of both mimotopes, and the control mimotope. **(C)** Jurkat T cells expressing mPD1 were used in a binding assay to examine the binding of anti-mPD1 mAb (10 ng/ml) alone or after preincubation with different concentrations of JT–mPD1 and the control mimotope. The inset shows reactivity of the anti-mPD1 mAb on Jurkat–mPD1 (gray histogram) and control Jurkat cells (open histogram) *n* = 3 for each data point. Significant differences are indicated by the *P*-values. **(D)** Flow cytometry histograms of a representative experiment are shown. Open histogram, no anti-mPD1 mAb; blue histogram, binding of the anti-mPD1 mAB; gray histograms, titration of the mimotope JT–mPD1, and the control mimotope. **(E)** Reporter gene (eGFP) expression of Jurkat hPD1 reporter cells activated by hPD–L1-expressing stimulator cells. The anti-hPD1 mAb (alone or after preincubation with JT–N1) was added as indicated *n* = 4 for each data point. The results are representative of at least two repeated experiments. Significant differences are indicated by asterisks (*P < 0.05, **P < 0.01, ***P < 0.001).

Jurkat reporter cells expressing hPD1 or mPD1 were employed to examine the capacity of the mimotopes JT–N1 and JT–N2, and also JT–mPD1, respectively, in inhibiting the binding of the corresponding mAbs to the respective cells. Examining the identified hPD1-derived mimotopes in a cell-based assay with Jurkat cells expressing hPD1, the mimotope JT-N1 was shown to significantly and more potently inhibit the binding of Nivolumab to the Jurkat cells, when compared to JT–N2 or a combination of JT–N1 and JT–N2 (Figure 2A). The specificity of the mimotope JT–N1 in inhibiting the binding of the mAb was further verified in the assay, by showing no binding inhibition of the mAb after pre-incubation with the control mimotope (Figure 2A). Flow cytometry histograms representing a binding experiment clearly reflected the specificity of mimotope JT–N1, compared to the control mimotope, in inhibiting the binding of Nivolumab to hPD1-expressing cells employed in the binding assay (Figure 2B).

The cell-based assay with Jurkat cells expressing mPD1 was also used for testing the capacity of the mimotope JT–mPD1, in comparison to the control mimotope, in inhibiting the binding of the corresponding mAb. As shown in Figure 2C, while no binding inhibition was caused by the control mimotope, preincubation of the mAb with the mimotope JT–mPD1 dose-dependently and specifically inhibited the binding of the mAb. The specificity of the mimotope JT–mPD1, compared to the control mimotope, in inhibiting the binding of the anti-mPD1 mAb was further illustrated by the flow cytometry histograms from a representative binding experiment involving the mPD1-expressing Jurkat cells (Figure 2D).

The hPD1-derived mimotope JT–N1 was further examined in an hPD1 and hPD–L1 reporter platform. While Nivolumab blocked the interaction between hPD1- and hPD–L1-expressing cells and consequently resulted in increased reporter activity, preincubation of the mAb with the mimotope dose-dependently and significantly inhibited the mAb's blocking capacity (Figure 2E), indicating the inhibitory capacity and specificity of the mimotope also in the reporter platform.

### Specific IgG Antibodies Raised Against JT–mPD1 in Rabbits Block the mPD1/mPD–L1 Interaction *in vitro* and Induce Anti-tumor Activity *in vivo* Following Passive Application

**Figure 3:**
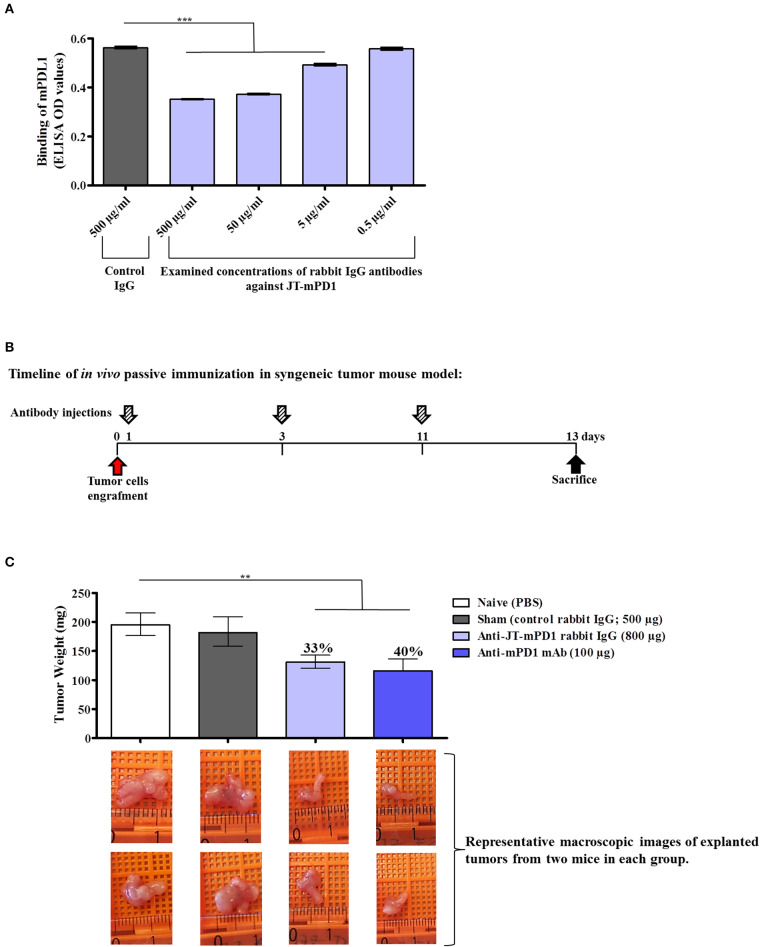
Evaluation of anti-tumor capacity *in vivo* by passive immunization with the rabbit IgG against JT–mPD1 in a syngeneic tumor mouse model. **(A)** Inhibition ELISA showing the binding of mPD–L1 to coated mPD1, before or after preincubation with control rabbit IgG or with different concentrations of rabbit-specific IgG against JT–mPD1 *n* = 4 for each data point. **(B)** BALB/c mice either remained untreated (naïve) or were injected as depicted. **(C)** *In vivo* anti-tumor effect shown by bars expressing the weight of the tumors explanted upon sacrifice from all the mice in each group. The levels of tumor growth reduction in the mice immunized with the rabbit IgG Abs or the examined mAb against mPD1, compared to the naïve mice, are indicated in percentages above the respective bars. Corresponding macroscopic images of representative explanted tumors are shown below each bar *n* = 8 for each data point. The results are representative of at least two repeated experiments. Significant differences are indicated by asterisks (**P < 0.01, ***P < 0.001).

To examine the capacity of the mimotope JT–mPD1 in inducing functional IgG antibodies, rabbits were immunized with the mimotope, specific IgGs were isolated, and the capacity of the antibodies to block the interaction between mPD1 and recombinant mPD-L1 was carried out by inhibition ELISA. As shown in Figure 3A, the examined JT–mPD1-specific rabbit IgG was shown to dose-dependently and significantly inhibit the binding of mPD–L1 to mPD1. These results indicated that the mimotope can potently induce IgG antibodies with a capacity in blocking the mPD1/mPD–L1 interaction in a dose-dependent manner.

The goal of immune checkpoint blockade in cancer immunotherapy is enhancement of T-cell activity to result in anti-tumor activity, as clinically proven in settings of passive immunization with immunomodulatory ICIs such as Nivolumab. Therefore, to examine whether the blockade of mPD1/mPD–L1 interaction by rabbit IgG against JT–mPD1 as shown *in vitro* can also be translated to anti-tumor activity *in vivo*, passive immunization/administration of the rabbit IgG in a syngeneic mouse model, involving BALB/c mice engrafted with BALB/c-derived mammary carcinoma (D2F2/E2) cells expressing human Her-2, was carried out. The D2F2/E2 tumor cells were used for grafting in naïve, sham treated, injected with JT–mPD1 rabbit IgG or anti-mPD1 mAb (positive control) BALB/c mice, as described in the Materials and Methods section and shown in Figure 3B. While similar weight of tumors in the naïve and sham-treated mice were observed, the rabbit IgG antibodies against the mimotope JT–mPD1 resulted in a significant tumor growth reduction (33%) in the immunized mice compared to the naïve mice. A significant tumor growth reduction of 40% was also observed in the tumors from the mice injected with the monoclonal anti-mPD1 mAb (Figure 3C).

### Active Immunization With mPD1-Derived Mimotope Leads to Significant Tumor Growth Reduction *in vivo*

**Figure 4:**
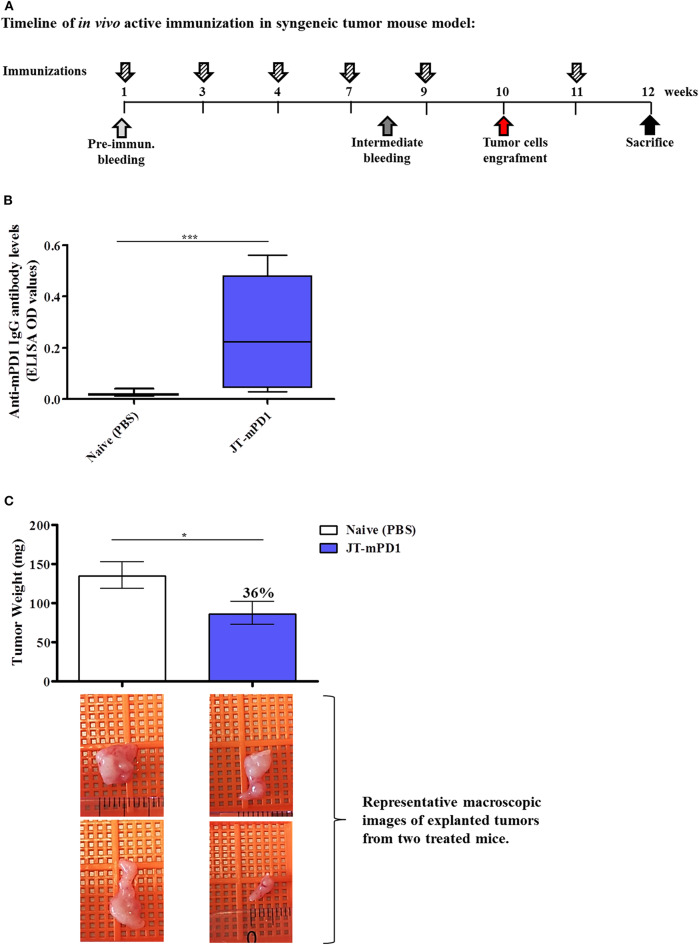
Evaluation of anti-tumor capacity *in vivo* by active immunization with JT–mPD1 in a syngeneic tumor mouse model. **(A)** BALB/c mice either remained untreated (naïve) or were immunized as depicted. **(B)** Level of serum IgG antibody responses against recombinant mPD1 protein, at the time of the sacrifice of the mice immunized with the mimotope. **(C)** *In vivo* anti-tumor effect shown by bars expressing the weight of the tumors explanted upon sacrifice from all the mice in each group. The level of tumor growth reduction in the immunized mice, compared to the naïve mice, is indicated in percentages above the respective bar. Corresponding macroscopic images of representative explanted tumors are shown below each bar *n* = 10 for each data point. The results are representative of at least two repeated experiments. Significant differences are indicated by asterisks (*P < 0.05, ***P < 0.001).

To evaluate whether active immunization with PD1-derived mimotopes induces an anti-tumor effect, the BALB/c mice, engrafted with the syngeneic tumor cell line D2F2/E2 expressing human Her-2/neu, were immunized with the mimotope JT–mPD1 (conjugated to CRM197 in conjunction with Montanide; 25 μg/dose) (Figure 4A). The mimotope was shown to be immunogenic and led to induction of mPD1-specific IgG antibody production (500 ng/ml, in average) in the immunized mice compared to the control mice (Figure 4B). As shown in Figure 4C, the induced antibody response was associated with a significant reduction (36%) of the tumor weight in the mice actively immunized with JT–mPD1, indicating a strong anti-tumor effect as a result of the active immunization with the mimotope, which was comparable to the tumor-reducing effects after passive immunization (Figure 4C).

### The Anti-tumor Effect by Active Immunization With mPD1-Derived Mimotope, but Not by Passive Immunization, Is Associated With Increased Cell Apoptosis (Cleaved Caspase-3) in the Tumors

**Figure 5:**
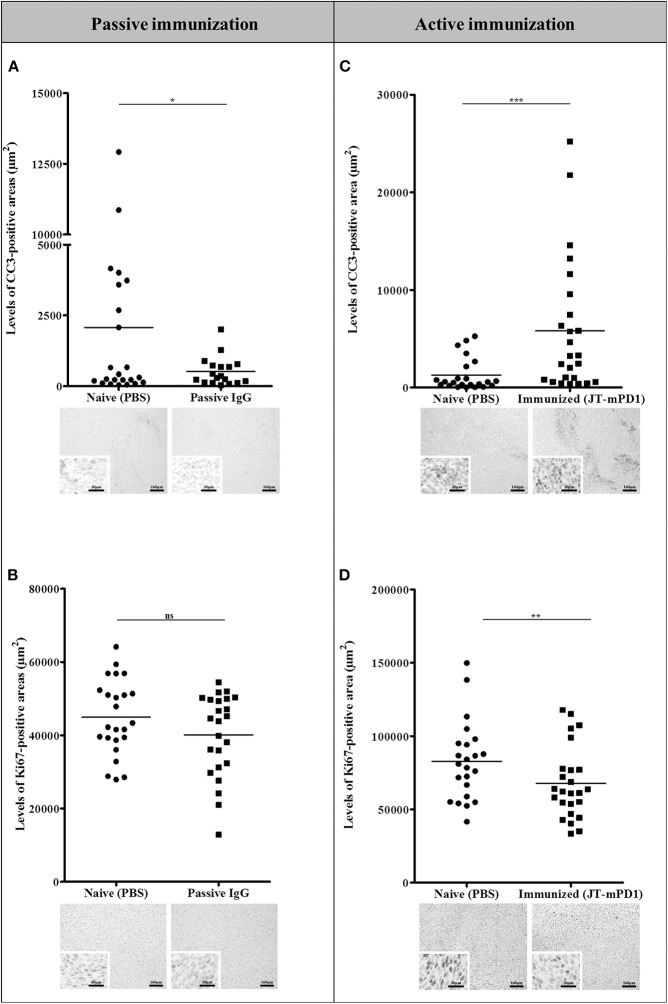
Comparison of the levels of the apoptotic marker CC3 **(A,C)** and the proliferation marker Ki67 **(B,D)** in the tumors of the mice passively immunized with the rabbit IgG against the mPD1-derived mimotope, or in the mice actively immunized with the mimotope, evaluated by IHC staining. For each tumor, more than one region was quantified for detection of evaluated markers. Representative images are shown. Significant differences are indicated by asterisks (*P < 0.05, **P < 0.01, ***P < 0.001). ns, not significant.

We further sought to evaluate the mechanism of the antitumor effect observed in the tumors from the passively immunized mice and those actively immunized with the mimotope JT–mPD1, by IHC for evaluating the levels of the cleaved caspase-3 (CC3; an established apoptotic marker and indicative of cell death) and of Ki67 (as a marker for cell proliferation). As shown in Figure 5, active immunization with the mimotope resulted in significantly increased levels of CC3 in the tumors, whereas no increase in the apoptotic marker was observed in the tumors from the passively immunized mice. However, in tumors from both the passive and active immunizations, decreased levels of the proliferation marker Ki67 were observed. These results suggested that active immunization, unlike the passive immunization, activates an apoptotic pathway in the tumor cells, although comparable levels of tumor growth reduction were observed after the two immunizations.

### Active Immunization With JT–mPD1 Does Not Lead to Signs of Increased Systemic Inflammation in the Mice

**Figure 6:**
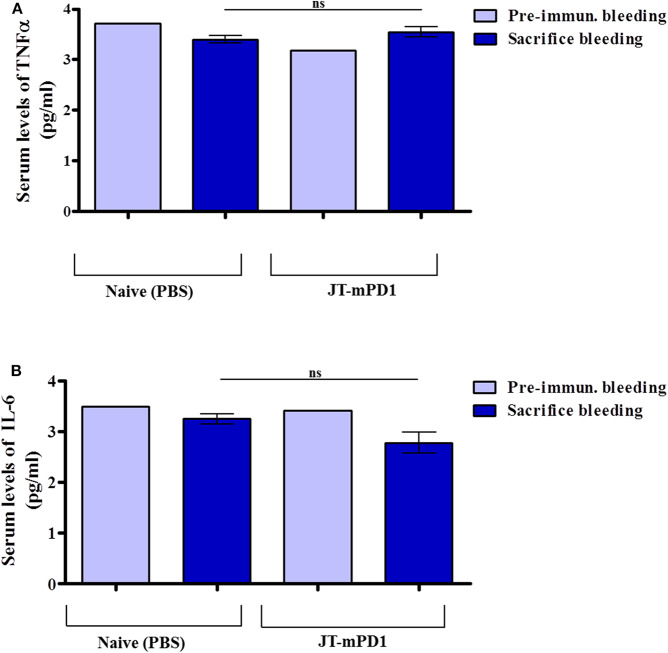
Detected levels of the pro-inflammatory cytokine tumor necrosis factor alpha (TNFα) and interleukin-6 (IL-6) in the sera of the mice actively immunized with JT–mPD1 compared to the naïve mice. The examined mice sera, prior to initiation of the immunization experiment (i.e., pre-immune bleeding), and from the sacrificed mice, were diluted 1/10, and the levels of the cytokines TNFα **(A)** and IL-6 **(B)** in the sera were examined by ELISA. The results are representative of at least two repeated experiments. ns, not significant.

To examine whether vaccination with the mouse-PD1-derived mimotope JT–mPD1 leads to elevated inflammation markers in the immunized mice, the pro-inflammatory cytokine levels TNFα and IL-6 were measured in the sera of the mice. Notably, no increase in the levels of the examined cytokines TNFα (Figure 6A) and IL-6 (Figure 6B) was detected, indicating that active immunization with the mimotope does not trigger systemic inflammation.

### Active Immunization Combining mPD1-Derived Mimotope Together With a Her-2/neu B-Cell-Based Vaccine (Her-Vaxx) Potentiates the Anti-tumor Effect *in vivo*

**Figure 7:**
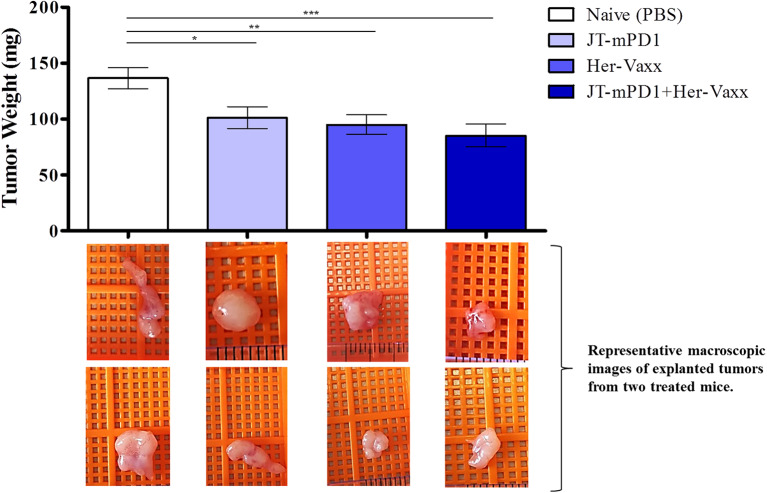
Evaluation of anti-tumor capacity by active immunization with JT–mPD1 in a syngeneic tumor mouse model. BALB/c mice either remained untreated (naïve) or were immunized, as depicted in Figure 4A, with mPD1-derived mimotope JT–mPD1, with a multiple B-cell epitope anti-Her2/neu vaccine (Her-Vaxx) or with combination of both antigens. *In vivo* anti-tumor effect shown by bars expressing weight of tumors explanted upon sacrifice from all the mice in each group. Corresponding macroscopic images of representative explanted tumors are shown below each bar *n* = 10 for each data point. The results are representative of repeated experiments. Significant differences are indicated by asterisks (*P < 0.05, **P < 0.01, ***P < 0.001).

Based on the above results, we next examined whether the mPD1-derived mimotope in combination with Her-2/neu vaccine could enhance the anti-tumor effect *in vivo* in the mouse syngeneic tumor model. Therefore, mice were immunized with either JT–mPD1 (50 μg/dose), our multiple B-cell epitope anti-Her2/neu vaccine (Her-Vaxx; 25 μg/dose) (22, 23), or a combination of both based on the schedule depicted in Figure 4A. As shown in Figure 7, immunization of mice with JT–mPD1 combined with Her-Vaxx significantly potentiated the anti-tumor effect compared to the effect seen in the mice immunized with each antigen alone.

## Discussion

Systemic administration of mAbs including immune checkpoint inhibitors targeting PD1, and their effect in immunomodulation, has demonstrated tremendous potential to control cancer growth in different tumor entities (8, 9). Following the concept of active immunization with B-cell-derived epitopes, as previously described against Her-2/neu (23, 24), in this study we describe the identification of PD1-derived mimotopes, and their use for active immunization to induce the host's immune system and inhibit tumor growth *in vivo*.

A surface-display platform for screening and detection of the mimotopes was established and applied, followed by a cellular platform for evaluating the effect of the identified mimotopes on the physiological interaction between T cells expressing PD1 and stimulator cells expressing PD–L1 (26). Among the identified hPD1-derived mimotopes, JT–N1 exhibited the strongest binding capacity to Nivolumab, specifically, and dose-dependently inhibited the binding of the mAb to recombinant hPD1 and also blocked the hPD1/hPD–L1 interaction in a T cell-based reporter assay *in vitro*. A high concentration (1,000 μg/ml) of the mimotope JT–N1 was required to achieve a significant level of inhibition. It has been described that Nivolumab binds to the N-terminal loop of human PD1 (29), where the identified and selected mimotope JT–N1 resides. It has also been reported that binding epitope of Nivolumab includes a few amino acids residing in the IgV domain of human PD1 (30). Therefore, it is conceivable that upon final folding, the N loop and the IgV region of the protein are in a proximity generating the optimal binding epitope of Nivolumab. By testing the overlapping peptides spanning the entire extracellular domain of human PD1, also including the IgV domain, only JT–N1 and the following overlapping peptides JT–N2 and JT–N3, which correspond to the N terminal side of the protein, were identified. JT–N2, which overlaps with JT–N1 by a few amino acids had a weaker capacity in inhibiting the binding of Nivolumab in the inhibition ELISA as well as in binding assay using a T cell-based cell line, whereas no inhibitory capacity was shown by JT–N3 in the assays. These results indicate the specificity of the mimotope JT–N1 and its importance in the region where Nivolumab binds to. However, we cannot exclude the possibility that the required high concentration of the mimotope may be due to missing residues required for the optimal binding of Nivolumab. Nonetheless, the mimotope JT–N1 has shown a strong and dose-dependent capacity in inhibiting the binding of Nivolumab in inhibition ELISA as well as in the cellular assays.

These results prompted us to prove whether immunization with PD1-derived mimotopes can reduce tumor growth *in vivo*. Consequently, a mimotope of an anti-mPD1 mAb with functional capacity was identified and examined *in vivo*. Using a syngeneic mouse model with mammary carcinoma tumor cells transfected for stable expression of human Her-2/neu, we showed that administration of IgG antibodies generated in rabbits against the mPD1-derived mimotope reduced tumor growth to the same extent as the administered corresponding mAb. These results indicated that the mimotope-specific antibodies have comparable biological activity as the corresponding monoclonal antibody. A similar observation of anti-tumor effect by passively administered anti-OX40 antibody in mice has been shown (31).

Importantly, the concept of active immunization with PD1-derived mimotope was proven in our syngeneic mouse model by showing that active immunization with the mPD1-derived mimotope can lead to reduced tumor growth *in vivo*. For this evaluation, the mimotope was conjugated to the carrier protein CRM197 and administered with the adjuvant Montanide, similar to our recently formulated B cell-based Her-2/neu vaccine (23). Our results showed for the first time that active immunization with the PD-1 mimotope induced significant anti-tumor effect compared to sham-immunized control mice. Analyses of the tumor growth reduction in the actively and passively immunized mice showed significantly increased levels of apoptotic marker CC3 in the mice tumors from the active immunization, while no increase was observed in the tumors from the passively immunized mice. These results clearly indicate that active immunization, unlike the passive immunization, activates an apoptotic pathway in the tumor cells. We speculate that active immunization may have promoted apoptotic cell death by inducing transcriptional expression of FAS ligand, which binds to FAS with subsequent caspase-3 activation, thus promoting apoptotic tumor cell death (32). Overall, the anti-tumor effect observed in the mice actively immunized with the mPD1-derived mimotope may not solely be linked to the capacity of the induced antibodies in inhibiting the interaction between PD1 and PD–L1, as the amount of antibodies induced by active immunization were clearly lower than the passively transferred antibody levels, indicating that active immunization induces broader immunological and cellular effects, in which pathways need subsequent in-depth analysis.

With regard to safety, it has been shown that treatment with immune-checkpoint inhibitors in humans can lead to inflammatory side effects/immune-related adverse events (33). In our model, we did not see any increase in the levels of the pro-inflammatory cytokines IL-6 and TNFα in the sera of mice actively immunized with the mPD1-derived mimotope, indicating that this immunization regimen does not lead to systemic inflammation. In addition, no apparent weight loss or other clinical signs of inflammation such as scrubby fur or deceleration of movement were observed during the course of the entire experiment. Nonetheless, further studies on long-term tolerability of active immunization, including co-sensitization with unrelated antigens or use of infections models, are currently planned to preclude potential harmful long-term effects on the immune system.

Several recent studies have focused on evaluating the anti-tumor effect of combined immunotherapy including cancer vaccine and ICIs, among which combinations of mAbs against human PD1 or PD-L1 were applied in conjunction with the clinically applied mAb Trastuzumab (34–38). Our *in vivo* experiments for evaluation of anti-tumor activity, involved the use of BALB/c mice-derived mammary carcinoma D2F2/E2 cells stably expressing human Her-2/neu. Therefore, we were prompted to examine the anti-tumor effect of active immunization with a combination of the mPD1-derived mimotope and our Her-2/neu vaccine (23, 24) in the syngeneic mouse model. Our results indicated an enhanced anti-tumor effect in the mice immunized with the combined vaccine compared to the mice immunized with each of the vaccines alone, suggesting that combinations of B-cell mimotopes derived from tumor-associated antigens and immune checkpoints possibly prolong and improve the efficacy of the respective cancer vaccines. As the concentration of the immune checkpoint-derived mimotopes can be optimized to a level showing highest efficacy with lowest side effects, they could serve as “adjuvants with good tolerability” to optimize the anti-tumor effects, highlighting the potential relevance of such mimotopes for clinical use. Furthermore, the use of mimotopes could overcome the limitations associated with direct use of monoclonal antibodies, including its cost-intensiveness for mono- or combination therapies and the linked adverse effects or possible development of resistance in some patients. *In vivo* studies are ongoing to examine the aspect, and this novel concept for treatment may be of particular value as treatment strategy in different tumor entities to prevent tumor recurrence (39–41).

Taken together, while the clinical benefit of antibodies targeting immune checkpoints is well-established, our results indicate that immune checkpoint inhibition can also be achieved by active immunization thereby providing a new concept for cancer immunotherapy. Immunization with mimotopes derived from immune checkpoints may not only contribute to the development of cancer vaccines using such mimotopes, as monovalent vaccines, but also pave the way for therapies combining tumor-specific antigens and thereby enhancing the efficacy of vaccinations against different malignancies adapted to the type, stage, and progression phase of the tumor.

## Data Availability Statement

All datasets generated for this study are included in the article/Supplementary Material.

## Ethics Statement

The animal study was reviewed and approved by the Animal Experimentation Committee of the Medical University of Vienna and the University of Veterinary Medicine as well as by the Austrian Federal Ministry of Science and Research (BMWF-66.009/0136-WF/V/3b/2017).

## Author Contributions

JT, PS, and UW: conception and design. JT, PS, and UW: development of methodology. JT, CB, AD, KB, KA, MD, SH, and LK: acquisition of data (provided animals, acquired and managed patients, provided facilities, etc). JT, CB, AD, SH, AI-K, LK, MK, PS, and UW: analysis and interpretation of data (e.g., statistical analysis, biostatistics, computational analysis). JT and UW: writing, review, and/or revision of the manuscript, writing and finalizing. CB, AD, ML, KB, KA, MD, SH, EG-S, MP, LK, MK, CZ, and PS: reviewing and commenting. KB, KA, and MD: administrative, technical, or material support (i.e., reporting or organizing data, constructing databases). JT, PS, and UW: study supervision.

## Conflict of Interest

MP has received honoraria for lectures, consultation or advisory board participation from the following for-profit companies: Bayer, Bristol-Myers Squibb, Novartis, Gerson Lehrman Group (GLG), CMC Contrast, GlaxoSmithKline,Mundipharma, Roche, BMJ Journals, MedMedia, Astra Zeneca, AbbVie, Lilly, Medahead, Daiichi Sankyo, Sanofi, Merck Sharp & Dome, Tocagen. The following for-profit companies have supported clinical trials and contracted research conducted by MP with payments made to his institution: Böhringer- Ingelheim, Bristol-Myers Squibb, Roche, Daiichi Sankyo, Merck Sharp & Dome, Novocure, GlaxoSmithKline, AbbVie; CZ was CSO of Imugene until June 2018. Consultancies and Speaker's Honoraria: Roche, Novartis, BMS, MSD, Imugene, Ariad, Pfizer, Merrimack, Merck KGaA, Fibrogen, AstraZeneca, Tesaro, Gilead, Servier, Shire, Eli Lilly, Athenex. Institution (CECOG): BMS, MSD, Pfizer, AstraZeneca; PS reports personal fees from Bristol-Myers Squibb outside the submitted work; UW was CSO of Imugene until September 2018 and has received from GSK, Pfizer and Themis study funding to the Institute. The remaining authors declare that the research was conducted in the absence of any commercial or financial relationships that could be construed as a potential conflict of interest.
