# Supplementary material for: A New Strategy Toward B Cell-Based Cancer Vaccines by Active Immunization With Mimotopes of Immune Checkpoint Inhibitors
Source: Front Immunol. 2020 May 27;11:895. doi: 10.3389/fimmu.2020.00895 (PMC7266955; doi:10.3389/fimmu.2020.00895)
Supplement: Supplementary file 1 [file Data_Sheet_1.pdf]

# Supplementary Materials and Methods

## Generation and expression of overlapping peptides

Protein sequences corresponding to the extracellular domains of mPD1 (Q02242; Uniprot) and hPD1 (Q15116; Uniprot) were electronically back-translated into DNA sequences (ATG:Biosynthetics GmbH; Merzhausen, Germany) and partitioned into uniformly-sized 15-mers overlapping peptides with 3-mers offsets. Expression vectors pEPX-1, containing DNA sequences encoding the 15-mer overlapping peptides from hPD1 and mPD1 downstream of Glutathione S-transferases (GST)-coding gene, Kanamycin as a selection marker and tac promoter regulated by lacI the product of *lacIq* were prepared (ATG:Biosynthetics GmbH; Merzhausen, Germany). Libraries with the expression vectors were prepared in separate pools, each containing 5-10 expression vectors with overlapping peptides from different regions of the corresponding protein. Each pool was used separately for electroporation of *E. coli* BL21, and the electroporated bacteria were recovered in SOC medium for 1 h at 37°C. Portions of the recovered bacteria were inoculated onto LB plates supplemented with Kanamycin (50 µg/ml), and the plates were incubated for overnight (O.N.) at 37°C. Five colonies per each expression vector were picked and streaked onto LB+Kan (master) and LB+Kan+IPTG (screening) plates. A clone harboring the expression vector with no insert, i.e. negative control, was also streaked on the screening plates. The plates were then incubated for O.N at 37°C.

## Colony blot assay for detection of clones expressing individual overlapping peptides of PD1

The screening plates with bacterial colonies from the electroporation steps were used for a colony blot assay (Dahlroth et al., 2006; Tobias et al., 2010). The bacterial colonies from the screening plates were replicated onto nitrocellulose membranes pre-soaked with Tris-HCl pH 8.0 50 mM, for 15 min at room temperature (RT). The membranes were then lifted and air-dried for 5 min at RT, with bacterial colonies facing upward. Subsequently the membranes were placed onto

a Whatman filter paper pre-soaked with lysis buffer (Tris-HCl pH 8.0 50 mM, 200 µg/ml Lysozyme), incubated at RT for 30 min, followed by three cycles of freezing at -80°C and thawing at 37°C. After the last step of thawing, the membranes were blocked with PBS- skim milk 2%. The blocking solution was discarded and the membranes were incubated for 2 h at RT with an employed anti-hPD1 monoclonal antibody (mAb) (Nivolumab, as an example) or with rat anti-mPD1 mAb with a blocking capacity (clone 29F.1A12; Biolegend, San Diego, CA), at the final concentrations of 10 ng/ml and 1 µg/ml, respectively, in PBS-Tween 0.05%-skim milk 0.5%. The membranes were then washed three times with PBS-Tween 0.05%, and incubated for 2 h at RT with the secondary antibody goat anti-rat IgG (mPD1; Santa Cruz Biotechnology) or goat anti-human IgG (hPD1; Sigma) conjugated to Alkaline Phosphatase. After additional washing cycle as above, the signals were developed using the substrate solution '1-Step NBT/BCIP' (Thermo Scientific, Rockford, IL).

**Sequence analysis**

Bacteria from the positive clones detected by colony blot were selected from the corresponding master plates and inoculated into 10 ml of LB medium supplemented with Kanamycin, followed by overnight incubation at 37°C and 180 rpm. A portion of each overnight culture was used for plasmid preparation using the 'GeneJET plasmid miniprep kit' (Thermo ScientificVilnius, Lithuania). The purified plasmids of the examined clone were mixed with a primer (5'-CATGGCCTTTGCAGGGCTGGC) annealing with the C terminal part of GST tag, and sent to Eurofins Genomics (Ebersberg, Germany). The retrieved sequences were aligned with the sequence of the corresponding proteins to identify the position of the encoded overlapping peptide in the examined positive clones. The expressed overlapping peptides in the positive clones were considered as mimotope candidates.

**Peptide synthesis**

Sequences of the identified mimotopes of the anti-hPD1 (JT-N1, JT-N2 and JT-N3) were sent for synthesis to GenicBio (China), and the synthesis and CRM197-conjugation of mimotope of anti-mPD1 mAb (JT-mPD1) was carried out at Provepharm Life Solutions (France).

**Immunization of rabbits with mouse PD1-derived mimotope**

Rabbits (n=2) were immunized at Charles River (France) according to a standard protocol: Subcutaneous immunization with 200 μg of the mouse PD1-derived mimotope JT-mPD1 conjugated to keyhole limpet hemocyanin (KLH), administered with Freund adjuvant, and additional three boosts with three weeks intervals. Total as well as mimotope-specific rabbit IgG were isolated at Squarix (Germany).

**Mixing of peptide with Montanide**

Montanide ISA-51-VG (Seppic, France), which is a water-in-oil emulsion, was used, as described previously (Tobias et al., 2017) for mice immunization with the mimotope JT-mPD1 and Her-Vaxx (a multiple B-cell epitope anti-Her2/neu vaccine) (Wiedermann et al., 2013).

**Detection of mPD1-derived mimotope-specific serum IgG**

Microtiter plates (Nunc Maxisorp, Denmark) were coated with recombinant mPD1 HIS-tagged protein (in PBS; 0.1 μg/well; R&D Systems, Minneapolis, MN), and ELISA was performed as previously described (Tobias et al., 2017). Mouse mAb (IgG1; Clone 7A11B1; Abnova) targeting mPD1 was used as standard to measure the levels of the anti-mPD1 IgG antibodies in the sera of the immunized mice. After blocking, diluted sera from the sacrificed mice were added. Bound IgG were detected with HRP-labelled rabbit anti mouse IgG antibody and subsequent TMB staining. Plates were read after adding stop solution at 450 vs 630 nm.

**Detection of cytokines in serum**

The levels of the pro-inflammatory cytokines IL-6 and TNFa in mice sera were examined using Ready-SET-Go! ELISA kits (eBiocience, San Diego, CA, USA).


Dahlroth, S.L., Nordlund, P., and Cornvik, T. (2006). Colony filtration blotting for screening soluble expression in Escherichia coli. *Nat Protoc* 1(1), 253-258. doi: 10.1038/nprot.2006.39.

Tobias, J., Holmgren, J., Hellman, M., Nygren, E., Lebens, M., and Svennerholm, A.M. (2010). Over-expression of major colonization factors of enterotoxigenic Escherichia coli, alone or together, on non-toxigenic E. coli bacteria. *Vaccine* 28(43), 6977-6984. doi: 10.1016/j.vaccine.2010.08.047.

Tobias, J., Jasinska, J., Baier, K., Kundi, M., Ede, N., Zielinski, C., et al. (2017). Enhanced and long term immunogenicity of a Her-2/neu multi-epitope vaccine conjugated to the carrier CRM197 in conjunction with the adjuvant Montanide. *BMC Cancer* 17(1), 118. doi: 10.1186/s12885-017-3098-7.

Wiedermann, U., Davis, A.B., and Zielinski, C.C. (2013). Vaccination for the prevention and treatment of breast cancer with special focus on Her-2/neu peptide vaccines. *Breast Cancer Res Treat* 138(1), 1-12. doi: 10.1007/s10549-013-2410-8.
